# An NB-IoT-Based Edge-of-Things Framework for Energy-Efficient Image Transfer

**DOI:** 10.3390/s21175929

**Published:** 2021-09-03

**Authors:** Sikandar Zulqarnain Khan, Yannick Le Moullec, Muhammad Mahtab Alam

**Affiliations:** Thomas Johann Seebeck Department of Electronics, Tallinn University of Technology, 19086 Tallinn, Estonia; yannick.lemoullec@taltech.ee (Y.L.M.); muhammad.alam@taltech.ee (M.M.A.)

**Keywords:** NB-IoT development platform, NB-IoT network, NB-IoT cloud, NB-IoT-based edge-of-things, image transmission

## Abstract

Machine Learning (ML) techniques can play a pivotal role in energy efficient IoT networks by reducing the unnecessary data from transmission. With such an aim, this work combines a low-power, yet computationally capable processing unit, with an NB-IoT radio into a smart gateway that can run ML algorithms to smart transmit visual data over the NB-IoT network. The proposed smart gateway utilizes supervised and unsupervised ML algorithms to optimize the visual data in terms of their size and quality before being transmitted over the air. This relaxes the channel occupancy from an individual NB-IoT radio, reduces its energy consumption and also minimizes the transmission time of data. Our on-field results indicate up to 93% reductions in the number of NB-IoT radio transmissions, up to 90.5% reductions in the NB-IoT radio energy consumption and up to 90% reductions in the data transmission time.

## 1. Introduction

Visual IoT is a paradigm where the environment is meant to be observed by camera-equipped IoT sensor nodes. The collected visual data from these nodes are transmitted to the cloud by means of adequate wireless communication technology. Several IoT communication technologies could be utilized for transmitting the collected visual data to the cloud and may include NB-IoT, LTE Cat-M1, LoRAWAN, Sigfox, etc. However, choosing a communication technology depends on the many factors that the particular technology has to offer in terms of its uplink/downlink data rates, transmission latency, device power consumption, as well as network availability and network coverage. Since visual IoT deals with transmitting a large amount of data to the cloud, it faces crucial challenges in terms of device power consumption, desired and achievable data rates, desired and achievable latency and the associated device and network cost.

**Figure 1:**
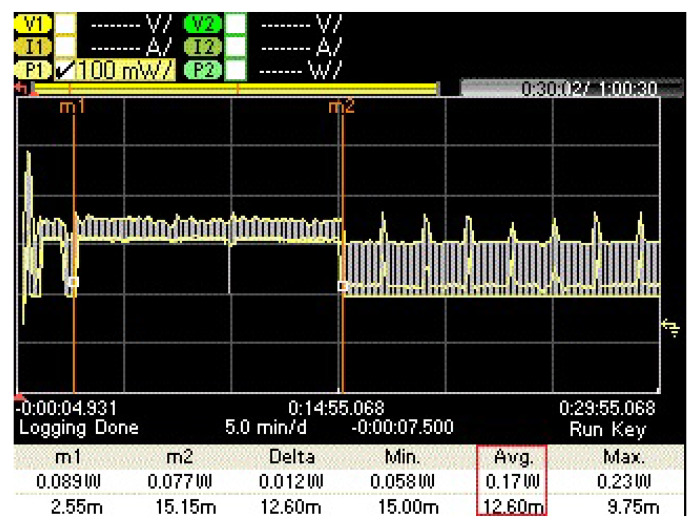
Power graph of the BG96 NB-IoT module when transmitting an image (1600 × 1200 pixels, 357.17 kB). A total of 239 transmissions are needed (12.60 m) with an average power of 0.17 W, which translates to an energy consumption of 0.0357 Wh.

In this work, we explore the suitability of NB-IoT technology for visual data transfer over the air with a focus on the channel occupancy, power consumption and time needed to transmit visual data from an individual NB-IoT radio. For illustration purposes, the power and time needed when using a naive implementation of visual data transfer from an NB-IoT radio is shown in Figure 1. The figure shows the power graph when transmitting a color image (1600 × 1200 pixels, 357.17 kB) over a Quectel BG96 radio module (in NB-IoT mode). Between markers m1 and m2, 239 transmissions are needed (corresponding to 12.60 m of channel occupancy) with an average power consumption of 0.17 W, which translates to an energy consumption of 0.0357 Wh.

While the transmit power of the NB-IoT module cannot be reduced, it is desirable to reduce the time and energy key performance indicators.

This raises several questions that to date have not been explored from a research point of view, which encompasses the following intertwined aspects:

- How should the overall NB-IoT architecture be organized for an efficient visual data transfer over the air? That is, how many hierarchical layers are needed and what could be their respective roles for collecting, processing, and transmitting data to the cloud?
- What are the suitable wireless communication technologies for transmitting visual data between the several layers of this architecture?
- What type of data processing is needed at what particular layer, taking into consideration the strength and limitation of each layer, and what could be the associated benefits in terms of the bandwidth utilization, channel flexibility and congestion alleviation?
- Lastly, what could be energy-latency trade-offs from the device and network perspective?

**Figure 2:**
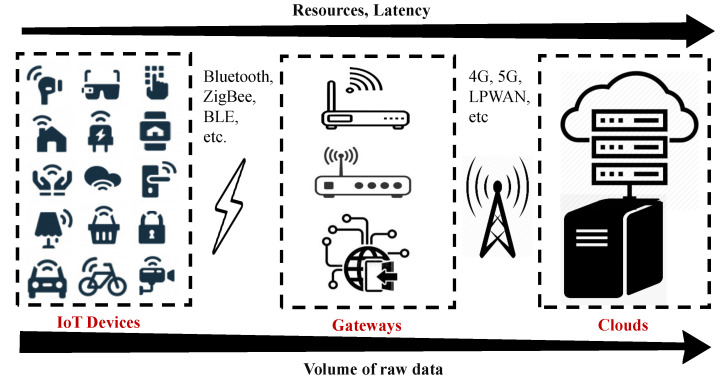
Hierarchical architecture of a generic gateway-based IoT testbed.

We address these questions by demonstrating a hierarchical smart-gateway-based visual NB-IoT testbed, as shown in Figure 2, where several heterogeneous IoT devices are connected to the gateway through short-range wireless communication technologies such as Bluetooth and Bluetooth low energy (BLE), and ZigBee, etc. The gateway is connected to the cloud by means of Low Power Wide Area Networking (LPWAN), specifically NB-IoT. The benefits of having a gateway-based system are many-fold. First, since IoT devices typically have low power budgets and limited computational and storage capabilities, the gateway-based setup allows these nodes to transmit their (visual) data to the gateway without any heavy computations. The compute-intensive gateway node thus performs all the heavy computations including running ML algorithms on the data that are to be transmitted over the air. Secondly, since only the gateway node provides access to the cloud through its LPWAN radio (i.e., NB-IoT in our case), the other nodes are only equipped with short-range communication radios i.e., BT, BLE. This minimizes the number of radios in the core (NB-IoT) network. Thirdly, since the gateway node is computationally capable, it carries out a substantial amount of local data processing, i.e., “Edge computing” for more control of data over the air. This can compensate for the limited bandwidth and lower data rates of LPWAN technologies, NB-IoT in particular [1]. We thus illustrate all these aspects through an edge-of-things computing-based NB-IoT framework for an efficient visual data transfer over the air and produce the associated on-field empirical results.

### 1.1. State-of-the-Art

C. Pham in 2016 (a year after the introduction of the LoRaWAN framework) used a LoRa network for the first time for image data transfer in a visual surveillance application [2]. He successfully transmitted an image of about 2.4 Kb up to 1.8 km using LoRa. Jebril et al. [3] proposed an approach for mangrove forest monitoring in Malaysia, wherein they transferred image sensor data over the LoRa physical layer (PHY) in a node-to-node network model. In their work, they also proposed a novel scheme for overcoming the bandwidth limitations of LoRa. Chen et al. [4] suggested a light trustworthy communication protocol called MPLR for image dispatching in LoRa to facilitate image monitoring in an agricultural IoT platform. Ji et al. [5] proposed a method for farming application wherein an image is transmitted into a tiny grid of patches such that any grid patch is only dispatched when a change in it is noticed. They showed that this approach reserves a lot of link budget during the surveillance of static agriculture sites and provides better performance. Wei et al. [6] proposed a methodology for transmission of JPEG compressed image data in a multiplexing mode with different spreading factors to reduce the transmission time of image data by keeping the quality of the images at the receiver side to high PSNR values. Other similar works that use LoRA for image transmission include [7,8,9].

From the perspective of utilizing edge computing for increasing the efficiency of IoT, several works have proposed and evaluated ML techniques for higher energy efficiency, bandwidth saving, lower latency, and collaborative intelligence of the network [1,10,11,12,13,14,15]. However, most of these works provide analytical models with simulation-based results that cannot be entirely relied upon for the real deployed networks because simulation-based validations do not accurately portray the empirical measurements of real-life systems. Other works such as [16,17,18,19] proposed deep learning for image recognition and classifications in IoT-based architectures. However, the focus of most of these works is mostly the obtained accuracy and precision of the models used for sending the final inferences to the cloud rather than original images. These works also lack the details on the amount of energy that is being consumed for ML computations with respect to the energy gains in terms of the device and network perspective. Nevertheless, some works proposed and utilized ML for increasing the energy efficiency of IoT nodes. For example the work in [20] uses an ML technique to determine whether to offload the classification of the current input data to the higher processing gateway layer or to perform it locally on the node and thereby achieve energy savings. However, they used the CC1350 IoT platform (a short range radio device) and lack details on larger families of networks such as LPWAN technologies. The work in [21] presented a hierarchical inference model to cut down the amount of data that are to be transmitted, and produced interesting energy-related results. However, they made use of BLE and ZigBee transmission protocols, lacking any correlation with LPWAN technologies. Though the work in [22] presented a real-time context-aware and collaborative intelligence among nodes in a large-area IoT testbed, they showcased their results using LoRa and BLE only. Other works that consider the use of ML for energy efficiency in LoRa networks include [23,24,25].

### 1.2. Contributions

The main contributions of this work and its positioning with reference to the state of the art and the aforementioned questions can be summarized as follows:

- We showcase a practical edge-of-things computing-based framework for dispatching optimized images over an NB-IoT test network wherein computations at the edge help reduce the number of NB-IoT radio transmissions over the core network.
- We practically show how the reductions in the communication budget of the radio can in turn contribute to relaxing the channel occupancy, minimizing the network load and reducing the transmission latency.
- We provide in-depth in-sensor analytics of the communication and computational cost of the gateway node along with mapping its energy-latency trade-offs.

The remainder of this paper is organized as follows. Section 2 provides an overview of the hardware architecture of our proposed three-layer hierarchical model and Section 3 presents the algorithmic structure at each layer. Section 4 presents in-field experimental results and energy evaluation of the proposed system. Finally, Section 5 concludes the paper.

## 2. Hardware Architecture of Our Proposed Three Layers Hierarchical Model

**Figure 3:**
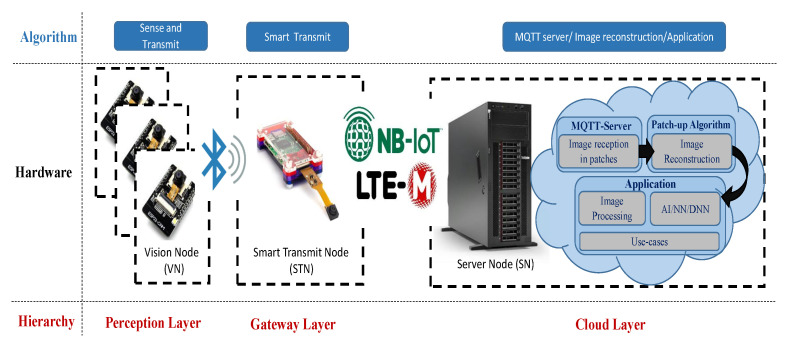
Proposed three-layer hierarchical model for energy-efficient image transfer via NB-IoT.

Our proposed system as shown in Figure 3 includes the following nodes at three hierarchical layers. 

A—Detection and Vision Node (DVN) at the Perception (Monitor) Layer;

B—Smart Transmit Node (STN) at the Gateway Layer;

C—Server node (SN) at the Cloud Layer.

### 2.1. Detection and Vision Node (DVN) at Perception Layer

**Figure 4:**
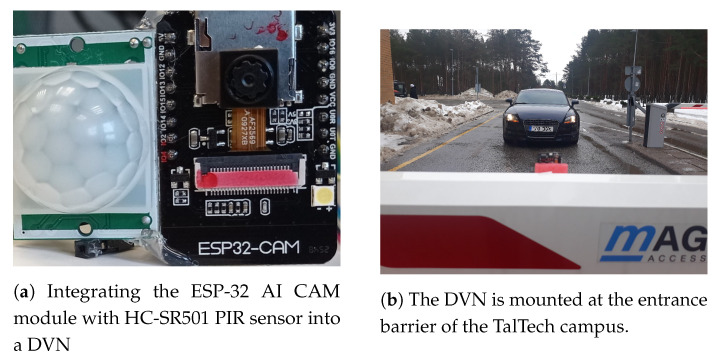
Detection and Vision Node at the perception layer.

An ESP32-CAM AI-Thinker module [26] is integrated with an HC-SR501 Passive InfraRed (PIR) sensor [27] into a Detection and Vision Node (DVN) that is mounted over the automatic gate barrier of Tallinn University of Technology (TalTech)’s main entrance as shown in Figure 4. The ESP32-CAM module is-based on an ESP32 SoC chipset [28] with Bluetooth/WIFI connectivity, a 2MP OV2640 image sensor, a built-in hardware JPEG encoder [29], and a support for power saving features. The HC-SR501 PIR motion sensor [27] can detect any motion within an adjustable range between 3 m and 7 m [30]. As any vehicle enters into the DVN’s sensitivity field, the PIR sensor detects its presence and wakes up the ESP-32 CAM module from its deep sleep mode. The ESP32-CAM module captures an image of the entering vehicle and sends it to the STN at the gateway layer through Bluetooth connectivity. As the image transmission ends, the DVN enters its deep sleep mode once again.

### 2.2. Smart Transmit Node (STN) at Gateway Layer

**Figure 5:**
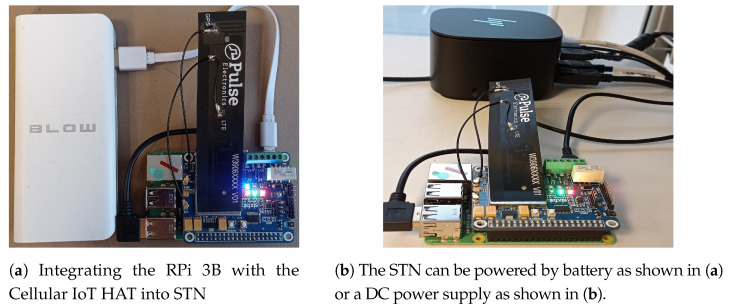
Smart Transmit Node at the gateway layer.

A Raspberry Pi (RPi) 3B module [31] is attached to an IoT cellular HAT [32] into a Smart Transmit Node (STN) and is deployed at the gateway layer of our proposed 3-layer hierarchical model as shown in Figure 5. The STN receives images from the DVN through a Bluetooth connectivity, processes it locally and smart transmits these images to the Server Node (SN) at the cloud layer through NB-IoT connectivity. The RPi 3B is preferred over its latest counterparts, i.e., RPi 3B+ and RPi 4B, as it has lower power consumption [33] and sufficient computational capability to safely run our ML algorithms. The attached Sixfab’s cellular IoT HAT is an add-on for Raspberry Pi that is based on Quectel’s BG96 chipset adding Cat NB1 (NB-IoT)/Cat M features to Raspberry Pi modules. More details on the module can be found in [34].

### 2.3. Server Node (SN) at Cloud Layer

Our physical server is based on an Intel Core-i7 platform that operates at 2.2 GHz, and is equipped with 8GB SRAM, 512GB HDD and runs Windows 10 as its Operating System (OS). Python (version 3.8) with all the required libraries runs on top of Windows 10. A Python-based HBMQTT broker (an open source MQTT broker and client implementation) is accessible to all its clients, both physical and virtual, through an internet connection via a dedicated IP address and a port.

## 3. Algorithmic Structure at Each Hierarchical Layer

The algorithms running across the various nodes of our three-layer hierarchical model include:

- A—A Sense a Transmit algorithm running over DVN
- B—A Smart Transmit algorithm running over STN
- C—An MQTT broker, Image reconstruction and an Application running over the SN

### 3.1. Sense and Transmit Algorithm over the DVN

**Figure 6:**
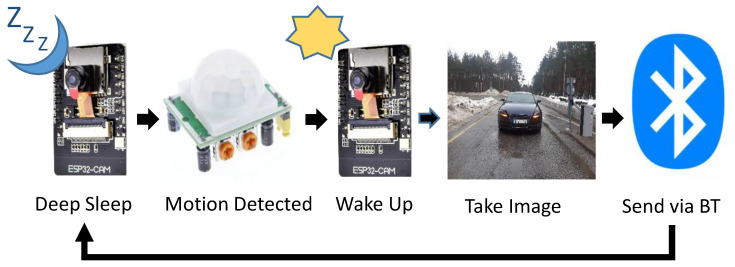
Sense and transmit procedure.

The algorithm running over the DVN works in a sense-and-transmit fashion where, upon any motion detection from the PIR sensor, the node wakes-up to capture an image of the approaching vehicle and sends it to the STN through a BT connectivity. As the transmission end, the DVN goes back into its deep sleep until triggered again by the PIR motion sensor. This is shown in Figure 6. The corresponding algorithm running over the DVN is given in Algorithm 1.
**Algorithm 1:** Sense and transmit algorithm: vehicle detection, image capture, and image transmission algorithm running inside the Detection and Vision Node (DVN).
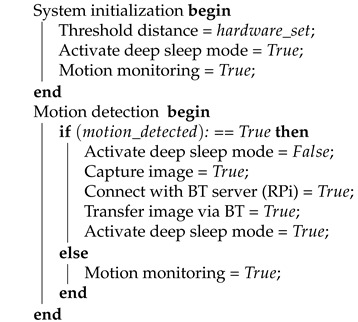


### 3.2. Smart Transmit Algorithm Running over the STN

**Figure 7:**
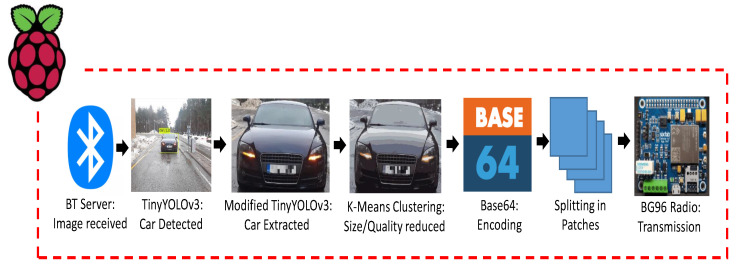
Smart transmit procedure.

The STN runs a series of algorithms in a sequential order as shown in Figure 7. They are discussed in their order of execution in the subsections to follow.

1—Bluetooth Server:

Bluetooth (BT) is capable of transmitting data wirelessly, in short range, at nearly 1 MB/s. Traditional BT works in a client–server architecture, such that the device that initiates the connection is called a BT-client (VDN in our case), and the one that accepts the connection is called a BT-server (STN in our case) [35]. As the BT-client attempts to initiate an outbound connection with the BT-server on the specified port, the BT-server establishes a two-way socket-connection if the connecting device is found to be authentic. Furthermore, the BT-server could connect to a maximum of 8 clients simultaneously at any particular time [35]. However, for test purposes, our BT-server connects to only one BT-client, i.e., the VDN that is installed at the entrance barrier of the TalTech campus and is situated at a distance of approximately 50 m from the BT-server. Upon connection establishment, the BT-server receives an image of the entering vehicle from the DVN and passes it to Tiny-YOLOv3 for vehicle detection and extraction as discussed below.

2—Tiny-YOLOv3 for vehicle detection and extraction: 

“You Only Look Once” (YOLO) is a state-of-the-art, real-time object detection algorithm proposed by Redmon et al. in their work in [36]. Since, traditional YOLO [37] is not suitable to run on embedded devices such as Raspberry Pi, due to its large memory size and high computational demands, a variation of the traditional YOLO called tiny YOLO-v3 [38] is installed to run on RPi 3B for vehicle detection in the received image. By modifying the code of tiny YOLO-v3, the detected vehicle in the image is extracted and saved as a separate JPG image file in the memory. Thus our modified tiny YOLO-v3 discards the unnecessary information from the received image that could produce significantly high costs both in terms of the RPi processing and NB-IoT radio transmissions (to be discussed later in the Results section). The output image from Tiny-YOLOv3 is then fed into K-means clustering for size reduction as discussed below.

3—K-MEANS Clustering algorithm: 

Since an image comprises a large data-set of pixels, such that each pixel is represented by 3 bytes that contain its RGB (Red–Blue–Green) intensity value in the range 0–255. Thus, K-means clustering could be exploited to cluster all the pixels of an image into similar RGB values and could be exploited for image compression [39], image segmentation [40] and color quantization [41,42].

For our particular use-case, we exploit the existing technique of [39] to use K-means clustering for image compression, i.e., by reducing the number of colors of an image to the most commonly occurring colors of an image. The number of k colors could be set as desired by the programmer or it could also be optimised to a minimum K that would output an image to a reasonable quality. It should be noted here that this method of compression gives significant reductions in terms of size of an image and leads to significant reductions in the number of radio transmissions. 

4—BASE-64 Encoding of the compressed image: 

Binary to text encoding schemes [43] become essential for transferring image data to web-sockets, so that the image data do not interfere with the many internet protocols in its way to its destination [44]. Moreover, Quectel’s BG96 module requires the use of the special character ‘CTRL+Z’ (by convention often described as ∧Z that is equivalent to the number 26 in decimals or 0x1A in hex) at the end of the data to be transmitted over the radio [45]. Thus, without using any encoding scheme, the radio cannot differentiate between the number 26 that might occur as part of the image data that are to be transmitted and the end of the image data [46]. Thus, the use of an encoding scheme becomes a necessity, especially when utilizing the BG96 module. Altogether, this is why we make use of the Base64 encoding scheme, the most commonly used encoding scheme, to transmit our image over the internet. 

**Figure 8:**
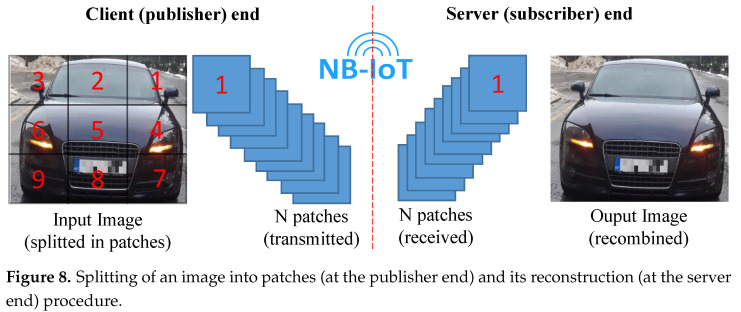
Splitting of an image into patches (at the publisher end) and its reconstruction (at the server end) procedure.

5—Transmission of the encoded image over the air through an MQTT protocol: 

The encoded image is sent over the air (NB-IoT network) by the BG96 radio utilizing an MQTT protocol [47]. Owing to its lightweight, low complexity, and easy implementation, the message queue telemetry transport (MQTT) protocol has become one of the most popular communication protocols for Machine-to-Machine (M2M) connectivity in the Internet-of-Things (IoT) paradigm [47]. The MQTT protocol works in a publish-subscribe fashion and runs on top of the Transmission Control Protocol/Internet Protocol (TCP/IP). The publish-subscribe mechanism requires a broker, also known as server, to which all the clients connect and share their information. A client that sends a message, through the broker, is called a publisher while a client that receives a message, through the broker, is called a subscriber. The broker filters all the incoming messages from all the publishers and distributes them accordingly to the subscribers. More details on the MQTT protocol architecture an its working can be found in [48]. Since the MQTT protocol supports sending a maximum payload of 1548 bytes [45], images of larger size cannot be transmitted in a single communication transaction. To overcome this, an algorithm running inside the STN breaks down each image into a grid of patches, each of 1500 bytes, such that these patches are dispatched separately with a header indicating its order in the source image. This is shown on the left side of Figure 8. The number of communication transactions that are required to send an image that is larger than the minimum transaction of ca. 1500 bytes size is given as Equation (1). Furthermore, the re-transmissions in NB-IoT (where up to 128 repetitions are possible in the uplink communication) ensures the successful reception of each segment at the receiver end, even in low coverage areas [49].

(1) $$\#\_communication\_transactions=\left\lceil \frac{image\_size\;(\mathrm{kB})}{1.5}\right\rceil$$

### 3.3. MQTT Broker, Image Reconstruction and an Application Running over the SN

A python-based HBMQTT broker [50] supporting a full set of MQTT 3.1.1 protocol specifications, runs over the SN and is accessible to any physical/virtual client through an IP address and a port. Since the image from STN is received in patches, a -Python-based image-reconstruction algorithm combines all these received patches in proper order to re-construct the encoded image as sent from STN.

The application, used as an example, monitors a parking lot in terms of the authorized vehicles. The collected images contain detailed information of the entering vehicles such as their type, color and more specifically their license plate numbers. These images are compared against a database of authorized vehicles. When an unknown vehicle is detected, the security services are notified with the visual description of the front of the vehicle. The image processing performed at the edge node (more specifically, the smart gateway) reduces the size of the images by (i) extracting the region of interest (front of the vehicle) and (ii) decreasing the number of colors. While (i) is bound by the contours of the front of the vehicles, (ii) is more flexible and we have explored various numbers of colors (K value in the K-Means algorithm) and identified that K = 12 is a good trade-off between reducing the image size while keeping sufficient quality regarding the license plate number and visual description of the front of the vehicle. In general, there could be a number of use-cases where image transmission would be required by applications such as [16,51,52], etc.

## 4. On-Field Experimental Trials with Energy/Time Consumption Evaluations

**Figure 9:**
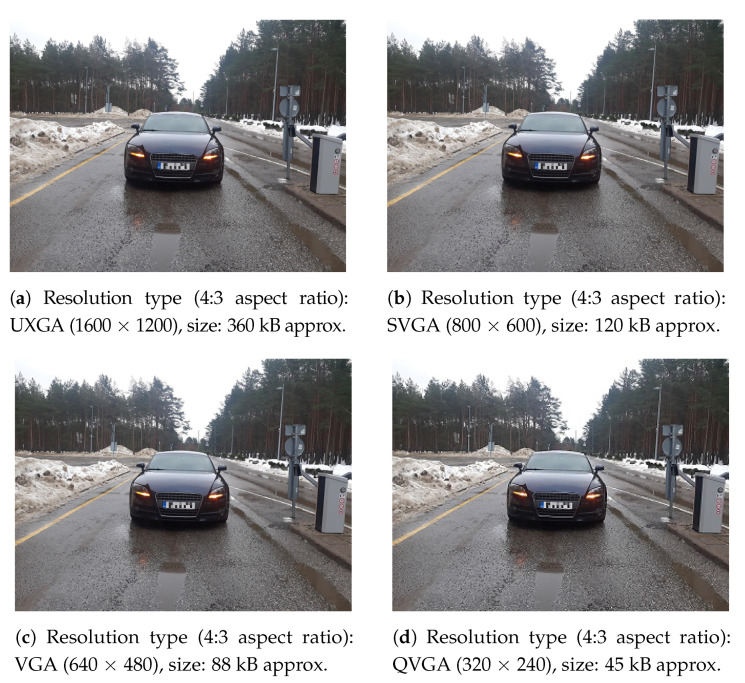
Images of different resolutions and sizes as generated by the field-deployed DVN at the entrance barrier of the TalTech Campus.

The field-deployed DVN is installed at the entrance barrier of Tallinn University of Technology (TalTech) campus and is configured to generate images of an approaching vehicle with different resolutions, i.e., (i) 1600 × 1200 full-resolution image, (ii) 800 × 600 medium-resolution image, (iii) 640 × 480, and (iv) 320 × 240 low-resolution images. Examples of these on-field images are shown in Figure 9 with their details given in Table 1.

**Figure 10:**
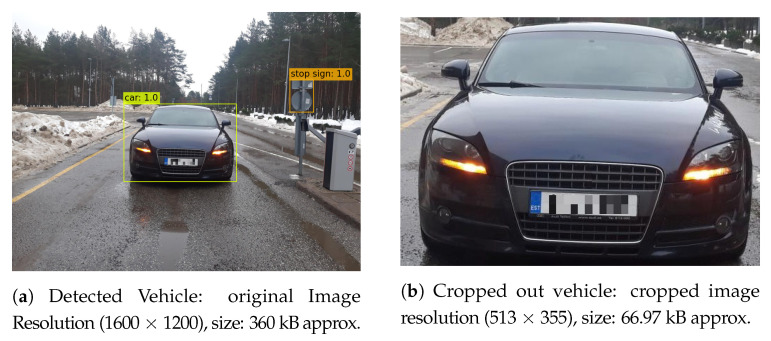
Output images from TINY-YOLOv3 algorithm: (**a**) detected vehicle and (**b**) croppedout vehicle.

The DVN after capturing an image of the vehicle transmits it to the GN through its Bluetooth connectivity. The received image at the GN is fed as an input image to the modified Tiny-YOLOv3 that extracts the detected vehicle and discards the rest of the unwanted information, i.e., bytes that could cause significant costs in terms of the communication and energy cost of the radio. Although this cropped-out image has a lower resolution as compared to its source image, its quality in terms of the number of associated colors remains the same. For example, for an input UXGA image (i.e., Figure 9a), the corresponding detected and extracted images are shown in Figure 10a,b, respectively. Since the output images from Tiny-YOLOv3 for the rest of images as shown in Figure 9b–d look the same, they are omitted from the display. However, their details are summarized in Table 2. For example, the first row in Table 2 indicates that an input image of 1600 × 1200 resolution and 357.17 kB size is reduced to an image of 513 × 355 resolution and 66.97 kB size, i.e., 81% reduction in the size of the source image thanks to TINY-YOLOv3 extraction.

**Figure 11:**
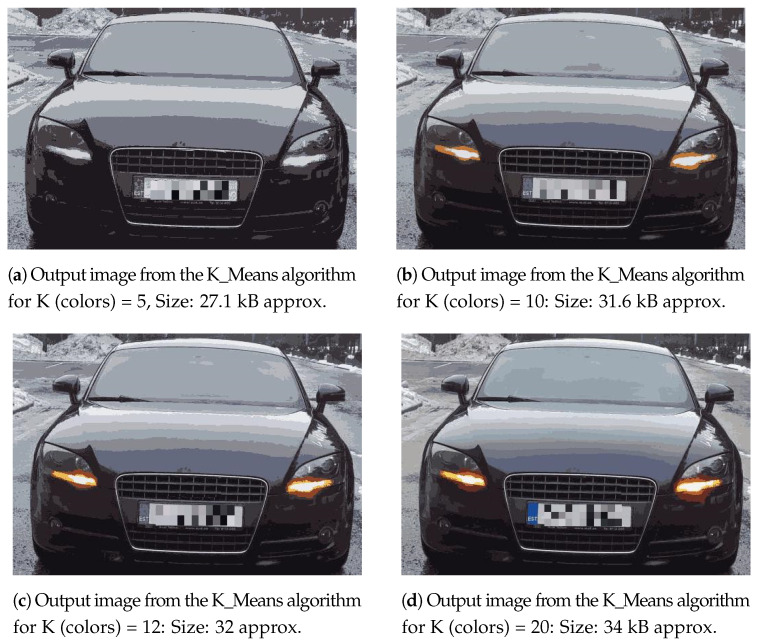
Output of the K_Means algorithm for an input image with a resolution of 513 × 355 pixels, total number of pixels = 182,115, total number of unique colors = 25,512 and an input size = 66.97 kB. (**a**) K = 5, (**b**) K = 10, (**c**) K = 12, and (**d**) K = 20. Note: the number plate in each output image from K_Means algorithm is mosaicked for display in this work for security reasons. However, the real application demands a visible number plate of the entering vehicle for identification.

The cropped-out image from Tiny YOLO-v3 is fed as an input to the K-means clustering algorithm for compression based on the reduction in its number of colors into K number of colors (i.e., K clusters). The output images from the K-means clustering algorithm for K = 5, 10, 12, and 20 with an input image of 513x355 resolution are shown in Figure 11a–d, respectively. For input images of other resolutions, the details in terms of their total number of colors, sizes and the corresponding output images from K-means clustering algorithm in terms of K number of colors and their resulting size(s) are summarized in Table 3. For example, the second column in Table 3 details the output images from the K-means clustering algorithm for an input image of 513 × 355 resolution for different K values. So, when it is in all of its 25,512 colors (output from Tiny YOLO-v3), it occupies 66.97 kB (as indicated by the third row and third column). However, when it is reproduced in only 12 colors (as indicated by the fifth row and third column), its size is reduced to 32.0 kB, almost half the original size. Thus, as the number of colors in the source image decreased (down the rows in first column), the corresponding size of the output images from K-means clustering algorithm decreases (in the corresponding size column).

The output image from the K-means clustering algorithm is then encoded into Base64 format for the reasons discussed in the previous section. After encoding, the image is split up into chunks, each of 1500 bytes, for possible transmission over the NB-IoT radio as a single transmission. The number of these chunks and their corresponding number of transmissions depend upon the total size of the image that is to be transmitted and can be derived from Equation (1).

### 4.1. Computation Cost

To assess the energy consumption of the proposed computations, i.e., execution of TINY-YOLOv3 followed by the execution of the K-means clustering algorithm, the power consumption of RPi 3B and its corresponding execution times for these algorithms were measured. It was found that the mean %CPU utilization of RPi was <10.0% in idle state, i.e., when no code was being executed while the mean %CPU utilization remained almost constant during the stress condition, i.e., above 90% during the execution of each individual implementation, i.e., TinyYOLOv3 and the K-Means clustering algorithmsIt was found that the mean %CPU utilization of RPi was <10.0% in idle state, i.e., when no code was being executed while its %CPU utilization reaches to a maximum of 93% in stress condition, i.e, when the code was being executed. It was also observed that the RPi’s CPU was never starved out even while processing the highest resolution image on a single core (note: for experimental purposes we disabled all but one of the cores to assess if it can handle the code with only one core). As for the current and power consumption, the Raspberry Pi 3B consumed, on average, a mean current of 260 mA at 5.0 V (which is about 1.3 W) in its idle state and it consumed, on average, a mean current of 350 mA at 5.0 V (which is about 1.75 W) under stress conditions. Table 4 summarizes the energy consumed by Raspberry Pi 3B for processing original images to create their optimized versions.

For processing an image, the RPi took, on average, 22 s to process a 2 MP full-resolution image (1600 × 1200) while it took, on average, 5 s to process the low-resolution (320 × 240) image. The RPi thus consumed, on average, 0.010 Wh of energy to process the high-resolution image while it consumed, on average, 0.0024 Wh of energy to process the low-resolution image. The per image energy consumption of RPi (i.e., computation energy) for processing of these images of various resolutions is summarized in Table 4.

### 4.2. Reducing the Communication Budget of an NB-IoT (BG96) Radio

Thanks to local computations (application of ML algorithms on images), the sizes of the (source) images are significantly reduced, as summarized in Table 3. These reductions in size contribute greatly towards minimizing the communication budget of an NB-IoT radio, both in terms of its number of transmissions and its energy consumption. Table 5 summarizes the total number of NB-IoT radio transmissions that are required to send images in their full resolution and colors (with no local computations) in comparison to sending their optimized versions with reduced resolutions and a reduced number of colors. For example, an input image of 1600 × 1200 resolution requires a total of 239 transmissions (3rd column) to be transmitted over the radio, while its optimized version image of 513 × 355 resolution 12 colors requires only 22 transmissions (6th column) to be transmitted over the radio, i.e., 90% reductions in the total number of NB-IoT radio transmissions.

To calculate the energy consumption of the BG96 radio (in NB-IoT mode) for the involved transmissions, the baseline energy consumption model for an NB-IoT radio as detailed in [53] is used, as represented by Equation (2). According to this equation, the total energy consumption of an NB-IoT radio is the sum of all of its energy states, i.e., Attach, Tx, Rx, TAU, eDRX and PSM where the energy consumption of each state is obtained by multiplying its power consumption parameter P_STATE_ by its timing parameter (T_STATE_) such that:

(2) $$\begin{array}{c} E_{TOTAL}=\\ \left(\begin{array}{c} \left\{P_{ATTACH(avg)}\times T_{ATTACH}\right\}+\\ \left\{\left(P_{Tx(avg)}\times T_{Tx}\right)+\left(P_{Rx(avg)}\times T_{Rx}\right)\right\}+\\ \left\{P_{C-DRX(avg)}\times \left(T_{InactivityTimer}\right)\right\}+\\ \left\{P_{TAU(avg)}\times T_{TAU}\right\} \end{array}\right)\\ +\left(\begin{array}{c} \left\{(P_{eDRX(avg)}\times T_{3324)}\right\}+\\ \left\{P_{PSM(avg)}\times \left(T_{3412}-T_{3324}\right)\right\} \end{array}\right) \end{array}$$

The sensitivity analysis (SA) of this model [53] is briefly discussed in what follows. First, Table 6 summarizes the power consumption parameters of this model in descending order and Table 7 summarizes the minimum and maximum values for the timing parameters of this model as standardized by 3GPP.

Then, since the power consumption parameters (Table 6) remain fixed for each state of the BG96 chipset, the timing parameters (durations) of these states (Table 7) affect the overall energy consumption of the radio to a greater extent. Considering the short periods of the Attach, and TAU states of the NB-IoT radio, their impact on the total energy consumption of the radio is not very significant. However, the transmission time (T_TX_) and the reception time (T_RX_) of the radio affect the overall energy consumption of the NB-IoT radio to a much greater extent as their duration prolongs. Thus, from the perspective of the sensitivity analysis of the NB-IoT radio, shortening the duration of its Transmission state (T_TX_) reduces its energy consumption to a greater extent and this is what we achieve through utilizing the ML algorithms at the STN node at the gateway layer, i.e., reducing the number of transmissions.

#### Communication Cost

**Figure 12:**
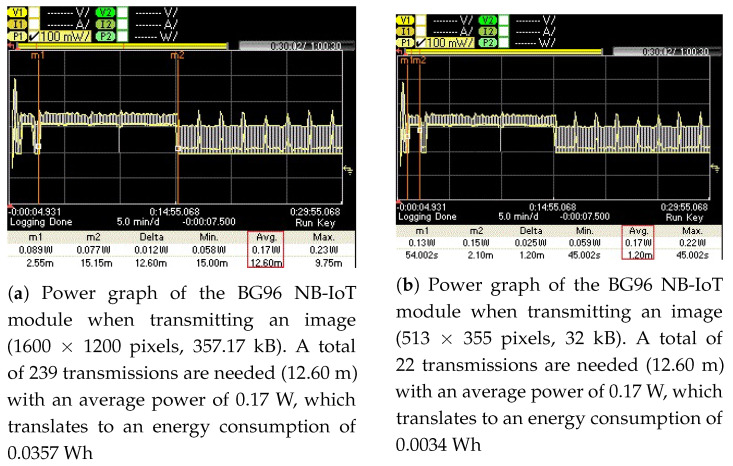
The energy consumed for transmitting an original image is (**a**) 0.0357 Wh and for transmitting its optimized version it is (**b**) 0.0034 Wh, i.e., 90.5% energy savings.

**Figure 13:**
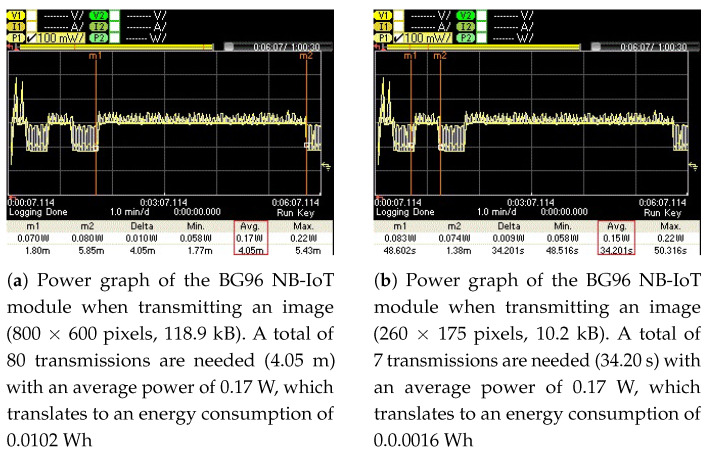
The energy consumed for transmitting an original image is (**a**) 0.012 Wh and for transmitting its optimized image it is (**b**) 0.0016 Wh, i.e., 84.3% energy savings.

**Figure 14:**
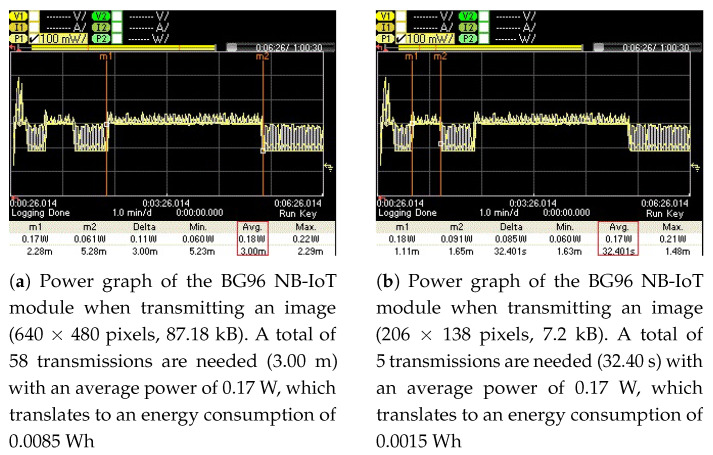
The energy consumed for transmitting an original image is (**a**) 0.0085 Wh and for transmitting its optimized version it is (**b**) 0.0015 Wh, i.e., 82.3% energy savings.

**Figure 15:**
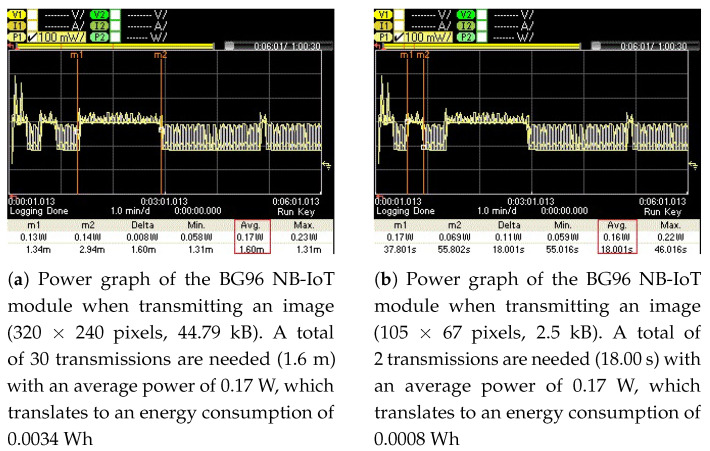
The energy consumed for transmitting an Original image is (**a**) 0.0034 Wh and for transmitting its Optimized image it is (**b**) 0.0008 Wh; 76.5% energy savings.

To measure the energy consumption of the BG96 radio (in NB-IoT mode) for transmitting these images (original and optimized) over the publicly available NB-IoT test network, their associated power graphs were measured using a Keysight Technologies N6705C DC Power Analyzer (PA) [54]. These power graphs are shown in Figure 12, Figure 13, Figure 14 and Figure 15 and display the average power consumption measurements of an NB-IoT (BG96) radio along with the associated transmission periods for the images, i.e., (a) when transmitted as original and (b) when transmitted as optimized. For example, Figure 12 shows that the BG96 radio consumed 0.0357 Wh of energy in transmitting the original image of 1600 × 1200 resolution while it consumed only 0.0034 Wh for transmitting its optimized version (with K = 12 colors). Figure 13 shows that the BG96 radio consumed 0.0102 Wh energy for transmitting an image of resolution 800 × 600 while it consumed only 0.0014 Wh of energy in transmitting its optimized version. Similarly, for Figure 14 the consumption of BG96 radio for transmitting an original image of resolution 640 × 480 is 0.0085 Wh where it consumes only 0.0015 Wh in transmitting its optimized version. Lastly, for Figure 15, the difference in the energy consumption of the BG96 radio for transmitting an original image of resolution 320 × 240 and transmitting its optimized version is 0.0008 Wh. Table 8 shows the transmission times of original images in contrast to the transmission times of optimized images. For example an original image of 1600 × 1200 resolution takes 12.6 min to be transmitted over the radio while its optimized version image of 513 × 355 resolution in 12 colors only take 1.20 min to be transmitted over the radio, a 90% in its transmission time as indicted by the third row of Table 8. Table 9 summarizes the energy consumption measurements for BG96 radio for transmitting original images in comparison to transmitting their optimized versions with K = 12 colors. The energy measurement calculation for each image is obtained by multiplying its average total transmission time (Figure 11, Figure 12, Figure 13 and Figure 14) with the transmission power of the radio, i.e., 0.17 W.

### 4.3. Trade-Offs in the Computation vs. Communication Costs

Table 10 summarizes the overall energy savings per image considering both their computation and communication cost. For example, when an original image of 1600 × 1200 resolution is transmitted from an NB-IoT radio (without any local computation) it consumes 0.0357 Wh (as indicated by the third row of Table 10). On the other hand, when it is processed locally by the RPi, it consumes 0.0107 Wh in computations (Table 4) and 0.0034 Wh in transmissions (Table 9), i.e., a total of 0.0141 Wh; this is a 0.0216 Wh energy difference, i.e., a total of 60% energy savings for a single image. Since Table 10 shows the energy savings per image; these individual energy savings scale-up as a multiple of the number of images that are processed by the RPi. For example, the RPi processed, on average, six images of 1600 × 1200 resolution in an hour, i.e., a total of 144 cars entered the campus in 24 h, and it thus saved on an average 0.20 Wh of energy in 24 h. With this rate, it can save on average 1.41 Wh of energy in a week and so on. It should also be noted that these energy savings are the outcome from a single STN at the gateway layer and these savings could further scale-up as a function of the increasing number of smart nodes in the network. As a side-note, since a single 100 W PVC solar panel generates around 400 Wh/24 h, a 15/20 W solar panel could also be utilized to power such a system [16].

**Figure 16:**
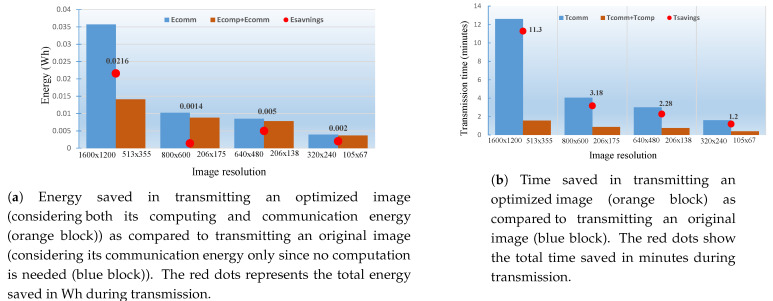
The energy and time saved in transmitting an optimized image of resolution 513 × 355 is 0.0216 Wh and 11.3 min. Similarly, energy and time saved in transmitting an image of resolution 206 × 175 is 0.0014 Wh and 3.18 min, and energy and time saved in transmitting an image of resolution 105 × 67 is 0.002 Wh and 1.2 min, respectively.

Finally, Table 11 summarizes the reductions in the transmission times for original images vs. the transmission times for optimized images. For example, an original image of 1600 × 1200 resolution takes 12.6 min to be transmitted over the air by a BG96 (in NB-IoT mode) radio while its optimized version (513 × 355 resolution in 12 colors) needs 22 s in computations and 1.20 min in transmission. This reduces the transmission time of this single image by 11.3 m, an 89% decrease in transmission time (i.e., 89% faster delivery) of the image. Figure 16 summarizes the energy and time savings in transmitting optimized images of 513 × 355, 206 × 175 and 105 × 67 resolutions as compared to transmitting their original counterparts of resolution 1600 × 1200, 800 × 600 and 640 × 480, respectively.

## 5. Conclusions

Our application verifies our proposed scheme for image transmission via NB-IoT, and this supports the industrial and academic trend which promotes NB-IoT as the future solution for IoT infrastructure.

Our on-field investigation showed promising results in terms of green Internet of Things, particularly ML for green and smart communications. Our results showed that potentially significant energy gains can be achieved by eliminating the unwanted data from transmitting over the IoT networks. We showcased this with our present setup operating on a publicly available NB-IoT network. We also showed that smart transmissions pays in terms of the increased responsiveness of the system.

Consequently, machine learning techniques running over the edge of any IoT infrastructure can potentially revolutionize the future IoT technologies that may include LoRAWAN, Sigfox, NB-IoT, CatM etc. The inference being done on the edge would save sending a huge amount of data over the IoT networks and would save significantly in terms of power, energy and timings of the over all IoT infrastructure.

In the future, we plan to optimize the operation of an NB-IoT radio in terms of its various operating states to improve its energy consumption, depending on the required latency and battery lifetime of a given application.

## Figures and Tables

**Table 1:** Image details with their resolution, aspect ratio, total number of pixels and their sizes in (kB) when stored in JPG format.

Resolution Type	Resolution (W × H)	Aspect Ratio	Max. No of Pixels (Max. 2MP)	Total Size (kB)
UXGA	1600 × 1200	4:3	1920,000	357.17
SVGA	800 × 600	4:3	480,000	118.89
VGA	640 × 480	4:3	307,200	87.18
QVGA	320 × 240	4:3	76,800	44.79

**Table 2:** Images with their input/output properties (resolution, size in kB) and percent reduction in size when processed in JPG format before and after the application of TINY-YOLOv3.

Input Image (JPG) Resolution	Detected Vehicle (JPG) Resolution	Input Image Size (kB)	Extracted Image Size (kB)	Percent Reduction in Size Thanks to Cropping
1600 × 1200	513 × 355	357.17	66.97	81%
800 × 600	260 × 175	118.89	20.51	82%
640 × 480	206 × 138	87.18	14.35	83%
320 × 240	105 × 67	44.79	4.60	89%

**Table 3:** K-means clustering on images of various resolutions, colors and sizes and the resulting images with K number of colors (clusters) and their output sizes in kB.

Images from T-YOLOv3	Resolution 1 (513 × 355)		Resolution 2 (260 × 175)		Resolution3 (206 × 138)		Resolution 4 (105 × 67)	
K Clusters	Color	Size	Color	Size	Color	Size	Color	Size
**K = all colors**	25,512	66.97	14,503	20.51	11,340	14.35	4830	4.60
K = 20	20	34.1	20	11.1	20	8.0	20	3.1
**K = 12**	12	**32.0**	12	**10.2**	12	**7.2**	12	**2.5**
K = 10	10	31.6	10	10.1	10	7.0	10	2.3
K = 5	5	27.1	5	8.2	5	5.8	5	2.1

**color**: No. of colors in the image, **Size**: given in kB. K = 12 (in bold font) corresponds to the best trade-off between the number of colors and achieved size reductions.

**Table 4:** Energy consumed by Raspberry Pi 3B for processing an original image to create an optimized version.

Per Image Energy Consumption of Raspberry Pi 3B			
Resolution	Computing Power (W)	Execution Time (s)	Energy Consumed (Wh)
1600× 1200	1.75	22	0.0107
800 × 600	1.75	18	0.0072
640 × 480	1.75	13	0.0063
320 × 240	1.75	6	0.0029

W: Watt, s: seconds, Wh: Watthour.

**Table 5:** Reduced numbers of transmissions for transmitting an optimized image with K = 12 colors in contrast to transmitting an original with K = all colors given in kB.

Original Image (Full Resolution, and All Colors)			Optimized Image (Cropped and K = 12 Colors)			R_ed__Tr (%)
Resolution	Size	R_eq_T	Resolution	Size	R_eq_T	
1600 × 1200	357.17	239	513 × 355	32.0	22	−90
800 × 600	118.90	80	260 × 175	10.2	7	−91
640 × 480	87.18	58	206 × 138	7.2	5	−91.3
320 × 240	44.7	30	105 × 67	2.5	2	−93.3

**R_eq_T:** required number of transmissions, **R_ed_Tr:** reduced number of transmissions, **R_eq_T:** is obtained by dividing the size of an image in kB by 1.5 as given in Equation (1).

**Table 6:** Power consumption parameters of the BG96 radio.

BG96 Power Consumption Parameters						
P_TAU_	P_ATTACH_	P_TX_	P_RX_	P_CDRX_	P_eDRX_	P_PSM_
0.18 W	0.18 W	0.17 W	0.16 W	0.083 W	0.070 W	0.0002 W

**Table 7:** Timings parameters of the BG96 radio.

Minimum and Maximum Values for the Timing Parameters				
T_TAU_/T_ATTACH_	T_TX_/T_RX_	T_CDRX_	T_eDRX_	T_PSM_
18.6 s (on avg.)	0 s–# of transmissions	10 s–60 s	0 s–186 m	0 s–413 d

**Table 8:** The reduced transmission period for transmitting an optimized image with K = 12 colors in contrast to transmitting an original image with K = all colors.

Reduction in the Transmission Period of NB-IoT Radio						
Original Image (All Colors)			Optimized Image (K= 12 Colors)			Red_TrTime (%)
Resolution	Size	TrTime	Resolution	Size	TrTime	
1600 × 1200	357.17	12.60 m	513 × 355	32.0	1.20 m	−90
800 × 600	118.90	4.05 m	260 × 175	10.2	34.20 s	−82
640 × 480	87.18	3.0 m	206 × 138	7.2	32.4 s	−82
320 × 240	44.7	1.6 m	105 × 67	2.5	18.0 s	−81

**TrTime:** transmission time (m: minutes, s: seconds), **Red_TrTime:** percent reductions in transmission time.

**Table 9:** The reduced energy consumption for transmitting an optimized image with K = 12 colors in contrast to transmitting an original image with K = all colors.

Reduction in the Energy Consumption of the NB-IoT Radio					
Original Image (in All Colors)		Optimized Image (in K = 12 Colors)		E_Red_ (Wh)	E_Red_ (%)
Resolution	E_con_ (Wh) *	Resolution	E_con_ (Wh)		
1600 × 1200	0.0357	513 × 355	0.0034	−0.0323	−90.5
800 × 600	0.0102	260 × 175	0.0016	−0.0086	−84.3
640 × 480	0.0085	206 × 138	0.0015	−0.0070	−82.3
320 × 240	0.0034	105 × 67	0.0008	−0.0026	−76.5

**E_con_:** energy consumed, **E_Red_:** energy reduction, **E_Red_ (%):** energy reduction in percentage, *: E_con_ is obtained by multiplying P_Tx_ with T_Tx_ (For reference see Table 6 and Table 7).

**Table 10:** Overall energy savings in transmitting an optimized image (considering both its computing and communication energy) as compared to transmitting an original image (communication energy only since no computation needed).

Energy Consumption per Original Image vs. Energy Consumption per Optimized Image					
Original Image		Optimized Image		E_Savings (Wh) (a–b)	ES (%)
Resolution	E_COMM_ (Wh) (a)	Resolution	E_COMP_ + E_COMM_ (Wh) (b)		
1600 × 1200	0.0357	513 × 355	0.0107 + 0.0034	0.0216	−60.50
800 × 600	0.0102	260 × 175	0.0072 + 0.0016	0.0014	−13.72
640 × 480	0.0085	206 × 138	0.0063 + 0.0015	0.007	−8.23
320 × 240	0.0034	105 × 67	0.0029 + 0.0008	0.002	−5.88

**E_COMM_:** communication energy, **E_COMP_:** computation energy, **E_Savings**: total energy savings per image, **ES (%)**: percent reduction in energy consumption.

**Table 11:** Overall time savings in transmitting an optimized image (considering both its computing and transmitting time) as compared to transmitting an original image (transmitting time only since no computation needed)).

Transmission Time per Original Image vs. Transmission Time per Optimized Image					
Original Image		Optimized Image		T_Savings (a–b)	TS (%)
Resolution	T_COMM_ (a)	Resolution	T_COMP_ + T_COMM_ (b)		
1600 × 1200	12.6 min	513 × 355	22 s + 1.20 min	11.3 min	−89.68
800 × 600	4.05 min	260 × 175	18 s + 34.2 s	3.18 min	−78.51
640 × 480	3.0 min	206 × 138	13 s + 32.4 s	2.28 min	−76.00
320 × 240	1.6 min	105 × 67	6 s + 18 s	1.20 min	−75.00

**T_COMM_:** transmission time, **T_COMP_:** computation Time(min: minutes, s: seconds), **ES (%)**: percent reduction in transmission time.
